# Identification of an Endothelial Senotype Predicted by Ion Channel Expression in Aged Murine Perigonadal Adipose Tissue

**DOI:** 10.64898/2026.10.26.761925

**Published:** 2026-10-28

**Authors:** Ann V. Hertzel, Syed Ali, Alessandro Bartolomucci, Jinhua Wang, Laura J. Niedernhofer, David A. Bernlohr, Christina D. Camell, Paul D. Robbins

## Abstract

Adipose tissue plays a key role in metabolic health, contributing to systemic dysfunction upon obesity or aging. A major driving factor of adipose-driven dysfunction is the accumulation of senescent cells (SnCs), but identification and characterization of specific SnCs is limited. To identify age-dependent SnCs, we performed snRNA-Seq on young and old adipose using two independent analytical strategies. The first, a SenMayo-based approach and the second, dependent on SEAC guidelines and Pearson correlation identified senescent nuclei in endothelial clusters. Subsequent subclustering and cell-to-cell communication analyses identified endothelial capillary nuclei that overexpress macrophage migration inhibitory factor (Mif), which exemplifies the specific characteristics and microenvironment of SnCs. Finally, rank analysis identified ion channel transport genes as the most predictive of the SnCs. Overall, these results highlight new defining features of senescence in endothelial adipose tissue and provide novel age-dependent biomarkers.

## Full Text Availability

The license terms selected by the author(s) for this preprint version do not permit archiving in PMC. The full text is available from the preprint server.

